# Identifying common core outcome domains from core outcome sets of musculoskeletal conditions: protocol for a systematic review

**DOI:** 10.1186/s13643-022-02120-1

**Published:** 2022-11-19

**Authors:** Tamer S. Sabet, David B. Anderson, Peter W. Stubbs, Rachelle Buchbinder, Caroline B. Terwee, Alessandro Chiarotto, Joel Gagnier, Arianne P. Verhagen

**Affiliations:** 1 Discipline of Physiotherapy, Graduate School of Health, The University of Technology Sydney, New South Wales, Australia; 2 Department of Health Professionals, Faculty of Medicine and Health Science, Macquarie University, New South Wales, Australia; 3 Center for Health and Wellness, Healthcare Department, Occupational Health, QatarEnergy, Doha, Qatar; 4 Sydney School of Health Sciences, Faculty of Medicine and Health, The University of Sydney, Sydney, NSW Australia; 5 Sydney Spine Institute, Burwood, New South Wales Australia; 6 School of Public Health and Preventive Medicine, Monash University, Victoria, Australia; 7 Department of Epidemiology and Data Science, Amsterdam UMC location Vrije Universiteit, Amsterdam, The Netherlands; 8 Amsterdam Public Health research institute, Methodology, Amsterdam, The Netherlands; 9 Department of General Practice, Erasmus MC, University Medical Center, Rotterdam, The Netherlands; 10 Department of Health Sciences, Faculty of Science, VU University Amsterdam, Amsterdam Movement Sciences, Amsterdam, The Netherlands; 11 Department of Epidemiology & Biostatistics, Schulich School of Medicine & Dentistry, Western University, London, Ontario Canada; 12 Department of Surgery, Schulich School of Medicine & Dentistry, Western University, London, Ontario Canada

**Keywords:** Outcomes, Domains, Core outcome sets, Musculoskeletal, Common outcome domains

## Abstract

**Background:**

Core outcome sets (COSs) aim to reduce outcome heterogeneity in clinical practice and research by suggesting a minimum number of agreed-upon outcomes in clinical trials. Most COSs in the musculoskeletal field are developed for specific conditions. We propose that there are likely to be common core domains within existing musculoskeletal COSs that may be used as a starting point in the development of future COSs. We aim to identify common core domains from existing COSs and to facilitate the development of new COSs for musculoskeletal conditions. As a secondary aim, we will assess the development quality of these COSs.

**Methods:**

A systematic review including musculoskeletal COSs. We will search Core Outcome Measures in Effectiveness Trials (COMET) database, MEDLINE, EMBASE, Scopus, Cochrane Methodology Register and International Consortium for Health Outcome Measurement (ICHOM). Studies will be included if related to the development of a COS in adults with musculoskeletal conditions and for any type of intervention. Quality will be assessed using the Core Outcome Set-Standards for Development (COS-STAD) recommendations. Data extracted will include scope of the COS, health condition, interventions and outcome domains. Primary outcomes will be all core domains recommended within each COS. We define a common core outcome domain as one present in at least 67% of all COSs. All findings will be summarized and presented using descriptive statistics.

**Discussion:**

This systematic review of COSs will describe the core domains recommended within each musculoskeletal COS. Common domains found may be used in the initial stages of development of future musculoskeletal COSs.

**Systematic review registration:**

PROSPERO CRD42021239141

**Supplementary Information:**

The online version contains supplementary material available at 10.1186/s13643-022-02120-1.

## Background

Musculoskeletal (MSK) conditions are a significant individual and societal problem representing a leading contributor to disability globally as identified in the Global Burden of Disease Studies [1]. MSK conditions such as osteoarthritis and rheumatoid arthritis can present with pain, movement limitation and loss of functional ability [2]. They are associated with increased healthcare utilisation, compensation costs, work-absenteeism and mental health impairments such as depression and anxiety [2–4]. This highlights the need to evaluate and improve upon existing management strategies for the prevention and treatment of MSK conditions. The World Health Organization (WHO) describes MSK conditisons as ‘[approximately] 150 diagnoses that affect the locomotor system; that is, the muscles, bones, joints and associated tissues such as tendons and ligaments’ as listed in the International Classification of Diseases [3].

The evaluation of the efficacy and effectiveness of management strategies for MSK conditions relies upon randomised controlled trials (RCTs) of low risk of bias and upon their synthesis in systematic reviews and meta-analyses. However, there is a lack of uniformity in outcome measurement across clinical trials in at least some MSK conditions [5] that significantly limits the capacity for researchers to combine and compare findings between studies. Additionally, the introduction of potential sources of bias, such as selective outcome reporting caused by reporting of favourable or statistically significant outcomes or the omission of unfavourable outcomes, is also known to impact the validity of the results of systematic reviews [6, 7]. Differences in comparable outcomes and the removal of relevant outcomes based on results can contribute to research waste by limiting the ability to pool results or to compare and contrast findings of RCTs [8, 9].

Accordingly, it is now recommended that core outcome sets (COSs) be used to reduce heterogeneity in outcomes measured across RCTs [10]. A COS is defined as an agreed minimum set of outcomes (or domains) that should be measured and reported in all RCTs for a particular health condition [11]. The use of COSs within RCTs increases the reporting of important and meaningful outcomes, reduces the risk of selective outcome reporting, increases the feasibility of pooling data in meta-analyses and improves their interpretation [8, 12]. Editors of Cochrane review groups agreed that the availability of COSs would enhance the validity of Cochrane reviews [6]. Finally, the use of a COS increases the feasibility of evaluating the “value” of interventions in RCTs. Value of healthcare, defined as the outcomes that matter to patients and the costs to achieve those outcomes, is of increasing interest to hospitals, funders and policymakers [13–15]. Outcome Measures in Rheumatology (OMERACT) and Core Outcome Measures in Effectiveness Trials (COMET) initiatives provide guidance on a process that includes patients and clinicians for reaching consensus on “what” should be measured (i.e., “outcomes” or “domains” or “outcome domains”) and “how” (i.e., "instruments" or "outcome measurement instruments") these should be measured within a COS [16, 17]. Following OMERACT, outcomes are identified using a three-tier system, whereby the core tier includes “mandatory domains”, the second tier includes “important but optional domains” and the third tier includes “research domains” [18]. These domains are chosen based on their importance to patients and clinicians. The process of developing a high-quality COS consists of various steps and might take years to develop. In recent years, several COSs have been developed for MSK conditions such as low back pain, shoulder pain and osteoarthritis [19–21]. Within these existing COSs, there is likely to be overlap in the included domains and common core domains may be identified that could be used as suitable candidate domains during the development phase of future MSK COSs.

Therefore, our primary aim is to identify and describe the overlap in core outcome domains within existing COSs for MSK conditions. The COSs evaluated will be those recommended for use in clinical practice and research to measure the outcomes of non-operative and operative interventions in adults. A secondary objective will be to assess the methodological quality of existing MSK COSs.

## Methods

The reporting of this protocol follows the recommendations of the Preferred Reporting Items for Systematic review and Meta-Analysis Protocols (PRISMA-P) statement and checklist [22]. Additionally, the definitions of key concepts and terminology used in this protocol closely follow those outlined by the OMERACT initiative [23]. The protocol has been registered at the International Prospective Register of Systematic Reviews (PROSPERO; registration number: CRD42021239141). Any amendments to the protocol will be described in detail within the final published systematic review.

### Eligibility criteria

A study will be considered eligible if it concerns the development of a COS including the terms “domain”, “outcome domain”, “outcome subdomain” or “outcome”. These terms are regarded as interchangeable and refer to a measured aspect of health “arising from exposure to a causal factor or health condition” [23]. A COS is defined as “the minimum set of domains and subdomains to be measured in clinical research or practice” [11]. These studies most often will be a mix of qualitative and quantitative processes, including surveys, Delphis, and consensus meetings. We will not restrict the type of study design to be included.

Using the definition proposed by the WHO and OMERACT initiative, for the purpose of this study, we have defined MSK condition to be “a situation of impaired health that affects the locomotor system; specifically, the muscles, bones, joints and associated tissues such as tendons and ligaments” [3, 23]. Rheumatic conditions are considered within the definition of an MSK condition as they directly impact the locomotor system affecting the muscles, bones, joints and associated tissues. Accordingly, studies will be included if they satisfy the disease categories for MSK conditions specified within the Global Burden of Diseases Study (2017) with described ICD 10 codes for “back pain”, “neck pain”, “rheumatoid arthritis”, “hip and knee osteoarthritis”, “gout” and “other MSK disorders” supplementary e-table 1, [24].

Additionally, COSs identified that are applicable to adults (greater or equal to 18 years old) and published in the English language or where a complete English language version is available will be included.

We will exclude any COSs describing a manifestation of what is not considered an MSK condition (e.g., pulmonary arterial hypertension related to systemic sclerosis would be excluded though systemic sclerosis would be included). Additionally, studies emphasising pain without providing details attributing pain to specific MSK condition or limb location will be excluded. Studies describing the International Classification of Functioning, Disability, and Health (ICF) Core Sets will also be excluded since they are not all-inclusive and do not include outcomes such as resource use and death [25]. Additionally, studies that limited scope of recommendations to only one core area (e.g., life impact only) or only to subdomains of a specific domain (e.g., pain) will also be excluded. Any studies seeking endorsement of an existing COS from stakeholder(s), or not specifying any core outcome domains, or studies assessing outcome measurement instruments rather than core domains will also be excluded. Finally, studies where full-text articles are not available or where only available by payment to commercial organisations/entities, or COSs identified as fact sheets, summaries and position statements without a description of methodology, will also be excluded as quality assessment cannot be performed properly.

### Study selection

Two review authors (TS, DA) will independently screen titles and abstracts for potentially eligible studies, with duplicates removed and ineligible papers excluded. They will then independently assess the full text of potentially eligible studies to determine final inclusion for the review. Consensus will be used to resolve any disagreement, but if an agreement cannot be reached, a third reviewer (AV) will be consulted.

### Search strategy

#### Electronic searches

All COSs listed in the COMET database [26] will be extracted using searched disease categories of: “anaesthesia & pain control”, “orthopaedics & trauma”, “rheumatology”, “rehabilitation” and “methodological and diagnostic” to include any publication within COMET published to the present date. For more recent COSs, not listed in COMET, additional eligible COSs will be identified using the multi-faceted search strategy described in the COMET annual systematic review [12]. The most recent update to the COMET database was for COSs published or indexed up to 2018 [26]. Accordingly, for studies not updated in the COMET database; biomedical databases MEDLINE (Ovid), Scopus (Elsevier), EMBASE (Elsevier), and the Cochrane Methodology Register will be searched from 1 January 2019 to 31 December 2021. The search strategy will combine index terms and text words to retrieve COSs for any health condition using a modified search strategy from Gargon and colleagues Appendix 1 [26].

#### Searching other resources

The International Consortium for Health Outcome Measurement (ICHOM) standard sets that meet the study eligibility criteria will be considered [27]. Further grey literature searching will be undertaken using search term “OMERACT” within the worldwidescience.org search engine given that this search term is required to be included in the title of all OMERACT developed COSs. In addition to database searching, forwards and backwards citation tracking of included studies will be performed [28].

### Data extraction

Using an electronic data extraction form, two independent researchers (TS, DA) will extract data for all eligible studies. The following data will be extracted: bibliographic (first author, year of publication), COS scope (use setting, health condition, target population, intervention), COS stakeholder profile and consensus method (participant group involved, geographical location of participant group, COS development method) and COS domains (by core area, outcome, and if described whether mandatory, optional or for further research). Additionally, we will note if there is reference to a study protocol and if domain definitions have been defined. Furthermore, outcome measurement instruments will be extracted for each domain if included within a COS. All disagreements between the review authors over the data extraction for studies will be resolved by discussion. If agreement cannot be reached, a third reviewer (AV) will be consulted.

### *Assessment of methodological qualit***y**

Assessment of the development quality will be undertaken using the Core Outcome Set-Standards for Development (COS-STAD) recommendations [29]. These standards focus on the principles of COS design, consisting of eleven minimum quality standards: scope specification (4 standards), stakeholders involved (3 standards) and consensus process (4 standards). Adherence to these methodological approaches is considered important in assessing whether a particular COS is well developed [29]. Each study will be appraised by two independent assessors (TS, DA) using “yes” (meeting the standard), “no” (not meeting the standard), and “unclear” (unclear whether the standard has been met). Any disagreements will be resolved by discussion. “Unclear” ratings will also be discussed to arrive at a consensus on whether it meets a standard. A third reviewer (AC, AV, CT or PS) will be consulted if agreement cannot be reached.

### Outcomes

Our primary outcomes are all core domains recommended within each COS. The secondary outcome will be to assess the development quality of these MSK COSs using the COS-STAD recommendations.

### Analysis

We will perform descriptive analyses. The characteristics of each COS (in terms of scope and methods) and the core outcome domains of each MSK COS will be summarised and presented. Each domain will be mapped to the respective core area and outcome domain using the Williamson/Clarke (initial) taxonomy [30]. We will use Cohen’s kappa to evaluate inter-rater reliability of COS-STAD rating. To identify common core outcome domains that are common within the MSK COSs, we propose that a core outcome domain must be present in 67% of COSs to be considered as common. This a priori threshold is considered in accordance with similar thresholds used within the development of individual MSK COSs [31, 32]. Sensitivity analysis will be undertaken to assess the effect of COS methodological quality on the selection of common core domains. The analysis of common core domains will prioritise COSs with high methodological quality. COSs will be considered to have high methodological quality if they meet all 11 recommended minimum standards of the COS-STAD. Extracted outcome instruments will be collated with the respective common core domain. All data will be stored using dedicated research data storage systems at the University of Technology Sydney.

## Discussion

This systematic review will identify and describe core outcome domains contained within existing COSs for MSK conditions. The development quality of these MSK COSs will be assessed and the results presented to identify common core outcome domains contained within studies with higher methodological development quality. We propose that the identification of core outcome domains will be useful in the initial phase of COS development for yet-to-be-developed condition specific MSK COSs. Specifically, this will be useful within the “What to Measure” stage of development recommended in the OMERACT and COMET frameworks [16, 17]. During this stage, researchers are usually required to identify existing potential candidate domains by using various methods such as reviews of literature (e.g., randomised controlled trials and/or qualitative studies). Following this, an appropriate consensus method is usually used to collect views of important outcome domains for the condition of interest. A generic MSK COS would allow the researchers to focus on identifying any candidate outcome domains that should be considered in addition to the MSK common core outcome domains identified in this study. It is anticipated that this will reduce research time in COS development processes that can often take many years. Finally, the assessment of development quality of the existing MSK COSs will also enable recommendations for future MSK COS development regarding any identified areas of improving methodological quality as related to scope, stakeholders, and the consensus process.

## Supplementary Information

**Additional file 1.** Appendix 1.

## Data Availability

Not applicable.
